# High‐resolution melting of the *cytochrome B* gene in fecal DNA: A powerful approach for fox species identification of the *Lycalopex* genus in Chile

**DOI:** 10.1002/ece3.5230

**Published:** 2019-06-14

**Authors:** Leonardo Anabalón, Francisco Encina‐Montoya, Pamela Sánchez, Jaime Solano, Felipe Benavente, Basilio Guiñez, Flavio Olivares, Carlos Oberti, Rolando Vega

**Affiliations:** ^1^ Departamento de Ciencias Biológicas y Químicas Universidad Católica de Temuco Temuco Chile; ^2^ Departamento de Ciencias Ambientales Universidad Católica de Temuco Temuco Chile; ^3^ Núcleo de Estudios Ambientales Universidad Católica de Temuco Temuco Chile; ^4^ Departamento de Ciencias Agropecuarias y Acuícolas Universidad Católica de Temuco Temuco Chile; ^5^ Facultad de Ciencias de la Salud Universidad Católica de Temuco Temuco Chile; ^6^ Corporación Nacional Forestal Temuco Chile; ^7^ Consultora Tierra del Sol Vallenar Chile; ^8^ Núcleo de Producción Alimentaria Universidad Católica de Temuco Temuco Chile

**Keywords:** barcode, HRM, *Lycalopex*, PCR

## Abstract

Easy, economic, precise species authentication is currently necessary in many areas of research and diagnosis in molecular biology applied to conservation studies of endangered species. Here, we present a new method for the identification of three fox species of the *Lycalopex* genus in Chile. We developed an assay based on high‐resolution melt analysis of the mitochondrial *cytochrome B* gene, allowing a simple, low cost, fast, and accurate species determination. To validate the assay applicability for noninvasive samples, we collected fecal samples in the Atacama Desert, finding unexpectedly one species outside of its known distribution range. We conclude that the assay has a potential to become a valuable tool for a standardized genetic monitoring of the *Lycalopex* species in Chile.

## 1 INTRODUCTION

Six species of foxes of the genus *Lycalopex* Burmeister, 1854 are found in South America, distributed from Ecuador to southern Patagonia (Ochoa, 2011; Zunino, Vaccaro, Canevari, & Gardner, 1995). *Lycalopex* is represented by three species in Chile: *Lycalopex culpaeus* (culpeo or Andean fox), the largest in size and distributed throughout Chile, except on the Island of Chiloé (southern Chile); *Lycalopex griseus* (gray fox) distributed from the Antofagasta to the Magallanes regions, including the Island of Chiloé; and *Lycalopex fulvipes* (Darwin's or Chiloé fox) the smallest of these foxes with distribution between the Nahuelbuta mountain range and the Island of Chiloé (Celis‐Diez, Ippi, Charrier, & Garín, 2011; D'elía, 2013; Muñoz‐Pedreros, Yañez, Norambuena, & Zuñiga, 2017). Currently, the conservation states of both *L. culpaeus* and *L. griseus* are of least concern (LC), while *L. fulvipes*, the only wild canid endemic to Chile, is in the “Endangered” (EN) category; this species is among the highest conservation priorities in Chile (Cofré & Marquet, 1999; Muñoz‐Pedreros et al., 2017).

The methods most commonly used for studying carnivores are direct methods like sightings, traps, carcasses, and review of collections; indirect methods traditionally used are odoriferous patterns, camera traps, response to vocalizations, trace transects and excreta or fecal collection (Gese, 2004). In studies of the behavioral ecology and feeding habits of carnivores, species are generally identified by the size, form, smell, and/or diet composition of the feces (Johnson, Aldred, Clinite, & Kutilek, 1981; Major, Johnson, Davis, & Kellog, 1980); however, this method of identification may be of limited use and lead to identification errors in areas where carnivores co‐occur (Bulinski & McArthur, 2000; Davison, Birks, Brookes, Braithwait, & Messenger, 2002; Farrell, Roman, & Sunquist 2000; Fernandes, Ginja, Pereira, Bruford, & Santos, 2008; Hansen & Jacobsen, 2006; Onorato, White, Zager, & Waits, 2006; Wittwer, 2009). Identification of hairs in the feces, ingested during self‐cleaning, has also been used; however, patterns may change in different carnivores, preventing the standardized use of this technique (Harrison, 2002; Onorato et al., 2006). The use of molecular methods, such as the bar code of mitochondrial DNA (mtDNA) and the genotyping of nuclear DNA, has made it possible to reduce errors in the identification of species, as well as to resolve phylogenetic relationships by revealing distribution patterns of wild canids. (Ochoa, 2011; Tchaicka et al., 2016; Torés, 2007) to the more recent use of barcode regions of *cytochrome B* (Behrens‐Chapuis et al., 2018; Fernandes, Costa, Oliveira, & Mafra, 2017; Jeon, Anderson, Won, Lim, & Suk, 2017; Wang et al., 2016; Yacoub, Fathi, & Mahmoud, 2013).

A noninvasive way of obtaining mtDNA is through fecal collection, since this does not imply capturing animals to gain, blood or other tissues as DNA sources (Torés, 2007).

High‐resolution melting (HRM) is a sensitive genotyping method, based on the thermal denaturation characteristics of the amplicons. This method has higher performance information, never achieved by classical DNA melting curve analysis. HRM is performed using a fluorescent, double‐stranded DNA dye that can be applied in fully saturating conditions (Wittwer, 2009). The amplicon is analyzed by gradual denaturation through increasing temperature and decreased fluorescence caused by the release of intercalating dye from the DNA. The melting temperature (*T*_m_) and specific shape of the melting curve result from the DNA sequence, GC content, and amplicon length, (Vossen, Aten, Roos, & Dunnen, 2009), so it is a useful technique for obtaining species‐specific genotypes. Due to the increased demand for rapid, economic, easy, high‐throughput genotyping analyses, there has been a considerable focus on HRM, which can detect sequence variants without the use of sequencing or hybridization procedures (Reed & Wittwer, 2004; Tindall, Petersen, Woodbridge, Schipany, & Hayes, 2009). Here, we present a fast, inexpensive, accurate, and sensitive technique for identifying different species of *Lycalopex* based on the analysis of fecal DNA by the mitochondrial *cytochrome B* gene using HRM techniques.

## 2 METHODS

Two hundred *Lycalopex* fecal samples were obtained in the Atacama Desert, northern Chile, between Lat. 26°31′29.07″ and 26°34′59.86″S, Long. 70°30′57.68″ and 70°20′45.27″W during September 2014 and February 2015. The search and collection of the feces were carried out along transects defined according to the geomorphological characteristics of the stream and then from the prospective terrain to the study area to identify potential habitats such as trails, caves, and bushes used by foxes. Twenty‐seven sampling stations were selected in scrub, rocky, and sandy environments, which were georeferenced and where camera traps were installed to constantly monitor places and target species. With this information, a map where each sampling point was plotted. For the collection of samples in the field, the feces were distinguished from those of other carnivores by their characteristic shape, excluding the white or dry appearance, to ensure to collect feces from foxes.

### 2.1 Sample collection

#### 2.1.1 References samples

Fresh frozen skin tissues from each of the taxonomically determined Chile‐inhabiting species *L. culpaeus*, *L. fulvipes*, and *L. griseus* were used to obtain reference DNA for the PCR end‐time and Real‐Time reactions with HRM.

#### 2.1.2 Unknown samples from feces

DNA was extracted from 200 fecal samples, with the Isolate Fecal DNA kit (Bioline), following the manufacturer's instructions. As input material we used an amount of 150 mg taken from initial sample of the external contour of the feces. The DNA concentration was estimated by standard fluorometric methods, using the Qubit 2.0 (Thermo Fisher Scientific) following the manufacturer's instructions. The integrity of the extracted DNA was checked by agarose gel electrophoresis in 0.8% agarose gel. The concentration of the DNA samples was normalized to 25 ng/µl to dilute potentially present PCR‐inhibitors and stored at −20ºC for further use.

### 2.2 End point PCR standardization of the *cytochrome B* gene

DNA amplification using end point PCR was performed using the Applied Biosystems™ SimpliAmp™, 0.2 µM forward primer, 0.2 µM reverse primer, and 1 µl of 25 ng/µl DNA. The primer pair used for the *cytochrome B* gene was: LC CYTB‐F: 5′TTCCAGCACCATCCAATATTTCCGC 3′ and LC CYTB‐R: 5′GGCGCCGTTTGCATGTATGTAACG 3′. (Quinga, 2012), using an initial denaturing step at 96°C for 4 min followed by 35 cycles of 96°C for 30 s, 66°C for 30 s, and 72°C for 30 s. The amplicons were electrophoresed in agarose gel 2% TAE 1X and stained with GelRed (Biotium). These primers amplify a region of the mitochondrial *cytochrome B* gene. For general specificity validation, we performed a primer BLAST search (Ye et al., 2012) confirming that the primers are specific for Lycalopex as indicated in Quinga (2012). Amplification tests were performed by end point PCR to standardize the reaction conditions. It is optimized according to the size of the amplicon.

### 2.3 High‐resolution melting (HRM) analysis

Real‐Time PCR was performed using the Eco Real‐Time PCR system (Illumina) in order to determine characteristic *T*_m_ of the different *Lycalopex* species to be distinguished. The reaction mixture for real‐time PCR and HRM analysis consisted of a total volume of 15 µl. This contained 7.5 µl of 2× Kapa SYBR qPCR Mix (Kapa Biosystems Roche), (Cowan & Elkins, 2018; Solano et al., 2019) 0.2 µM forward primer, 0.2 µM reverse primer, and 1 µl of 25 ng/µl DNA. SYBR fluorescence dye was used to monitor the accumulation of the amplified product during PCR and the HRM process to derive the *T*_m_ value. The same primer pair and PCR program as for end point PCR was used (Quinga, 2012).

Fluorescence data were measured at the end of each extension step during the PCR cycles. Subsequently, the PCR amplicons were denatured for HRM at 95°C for 15 s, and then annealed at 50°C for 15 s to form a random DNA duplex. The RT‐PCR HRM protocol collected fluorescence data at 0.1°C temperature increments. For the HRM analyses, the fluorescence data were collected in 0.1°C increments between 78 and 82°C. No cross‐species tests were used, since the primers were specific for the genus *Lycalopex*.

We used the EcoStudy Software v 5.0 to plot a normalized curve of decreasing fluorescence with increasing temperature. The negative derivative of the fluorescence (*F*) over temperature (*T*) (*dF*/*dT*) curve gives the *T*_m_. Three control DNA samples were obtained from fresh tissues of taxonomically identified *Lycalopex* species. To generate normalized melting curves and difference melting curves, pre‐ and postmelt normalization regions were set to define the main temperature boundaries of the normalized and difference plots in these controls, which were set as the species references. The control samples were provided by government agencies.

### 2.4 Sequence analysis

PCR products were directly sequenced in two directions for each product using a BigDye Terminator V3.1 Cycle Kit in an automated Genetic Analyzer 3500 xl (Applied Biosystems, HITACHI), with software Data Collection v3. Sequences were aligned and proofread using the software MEGA 5 and submitted to GenBank.

## 3 RESULTS

### 3.1 End point PCR standardization of the *cytochrome B* gene

Of the 200 DNA samples extracted, 150 were successfully amplified. These 150 samples were used for the HRM analysis. The samples analyzed were amplified approximately to a 200‐bp product, as previously reported for the *cytochrome B* gene (Yacoub et al., 2013) (Quinga, 2012). (data not shown).

### 3.2 Species determination of reference and unknown samples using HRM analysis of *cytochrome B* gene

**Figure 1:**
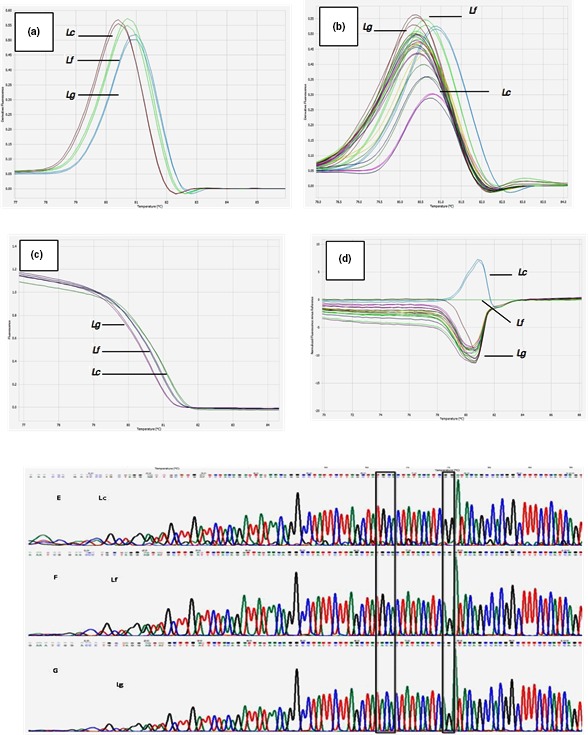
(a) Melting curves derived from reference samples; (b) Melting curves derived from fecal DNA plus reference samples; (c) Melting curves normalized from reference samples; (d) Difference graph. *Lycalopex fulvipes* is used as a reference; (e) Electropherogram of sequences of *cytochrome B* gene in: (e) *Lycalopex culpaeus*; (f) *L. fulvipes* and (g) *Lycalopex griseus*

Melting curves (Figure 1a,b) were generated by plotting the fluorescence against the temperature. Fluorescence declines as the DNA melts. DNA melting is visualized through the use of a saturating duplex‐dependent DNA intercalating dye. As the DNA melts, the dye is released; unbound dye does not fluoresce. The normalized curve (Figure 1c) was generated by taking the negative derivative of fluorescence with respect to temperature and plotting these values against temperature (−*dF*/*dT* vs. *T*). When *L. fulvipes* is selected as reference (Figure 1d) the obtained difference graph can clearly be distinguished by variation in peak position, positive peak for *Lycalopex culpeus,* and negative peak for *L. griseus*, shape change, and *T*_m_.

PCR of fresh tissue (reference samples) and fecal samples was successfully amplified; in both cases, it was possible to obtain amplifiable DNA for the region of interest. This is very important, since the conservation status of feces is often bad, especially in extreme environments like the Atacama Desert. In some cases of fecal samples, the amplification is not appreciated, precisely because of the presence of inhibitors elements in the DNA samples. Despite this difference in the amplification products, the temperatures and shapes of the melting curves for each amplicon are maintained.

The DNA fusion profile was analyzed to investigate whether there is a polymorphism in the *cytochrome B* gene of different fecal samples of the *Lycalopex* genus. This variation was clearly detectable in the derived and normalized melting curves (Figure 1a–d).

The obtained HRM curves could be categorized into three different characteristic peaks in the range between 80.4ºC and 80.9ºC. (*L. culpaeus* 80.9ºC, *L. fulvipes* 80.6ºC, *L. griseus* 80.4ºC), this values did not vary within a species, hence, there were no deviations in Tm detectable. All profiles of *Lycalopex* fecal samples produced only one peak. The HRM melting curves were different for each species of the *Lycalopex* genus. The samples were genetically distinguishable and therefore corresponded to the different genotype.

To confirm the identity of the specimens, the three species under study were sequenced and aligned with sequences retrieved from NCBI. Our results were also confirmed by sequence analysis of *cytochrome B* amplicons, which revealed that all three species of foxes were present in this analysis (*L. culpaeus, L. griseus* and *L. fulvipes*).

## 4 DISCUSSION

Significant improvements have been reported based on fecal DNA analysis for both extraction and quantification of nucleic acids as part of genotyping and species identification (Kanthaswamy, Premasuthan, Ng, Satkoski, & Goyal, 2012). Molecular analysis of feces is a noninvasive method with a high degree of integrity and quality when using specific commercial kits for these complex matrices (Deshpande, Villarreal, & Mills, 2016; Ramón‐Laca, Soriano, Gleeson, & Godoy, 2015; Tende, Hansson, Ottosson, & Bensch, 2014). The main challenge of this study was to obtain DNA of good quality from fecal samples collected in the Atacama Desert. The DNA samples obtained from these matrices were used to obtain information about the presence and distribution of *Lycalopex* species in the Atacama Desert, which is of great importance for promoting and orienting conservation measures.

Feces contain epithelial cells from the intestine, mucus, and hairs. DNA can be extracted from this material and amplified by PCR. Although there are previous studies that have used mtDNA from feces (Chaves, Graeff, Lion, Oliveira, & Eizirik, 2012; Quinga, 2012; Ray & Sunquist, 2001; Taberlet, Waits, & Luikart, 1999), there are no reports of the use of mtDNA to analyze the presence and distribution of *Lycalopex* species in Chile and South America. We developed an assay based on HRM analysis of the mitochondrial *cytochrome B* gene, used for identifying genetic variations in nucleic acid sequences. We also describe the HRM method for genotyping *Lycalopex* species, which shows the potential of the mitochondrial *cytochrome B* gene DNA barcode coupled with the HRM method to provide a fast and very accurate method of taxonomic identification and species identification.

We were able to identify all the three species of *Lycalopex* foxes inhabiting Chile using the developed assay; this is the first report of the presence of *L. fulvipes* foxes in northern Chile. The Chilean species of this genus are in different conservation categories. There are no reports on hybridization between species of the *Lycalopex* genus, only observations and stories of local people in the field, which refer to the possible hybridization without viability of the offspring. The melting curves obtained from amplified fecal DNA samples are grouped or positioned with respect to the curves obtained from the control reference samples of the taxonomically determined species (three species of the *Lycalopex* genus that live in Chile). We can also add that our evidence indicates that an approximate proportion of 60% was obtained for *L. griseus* and approximately 40% for *L. culpeus*. (Medel & Jaksic, 1988).

Surprisingly, we could detect one *L. fulvipes* sample, which was not expected to be present in our study area. This is the first report of the presence of foxes of *L. fulvipes* in northern Chile. Therefore, it is important to determine their presence and distribution in different ecosystems throughout the country. Although the presence of *L. fulvipes* may be less frequent in these latitudes, the deepening of monitoring of *Lycalopex* species in northern Chile is very important because due to possible unknown distribution ranges of the *Lycalopex* species. A possible cause for migrations into new habitats could be a response to climate change or events such as forest fires, increasingly common in southern Chile and specifically in the ecosystems where *L. fulvipes* lives.

There are no previous reports of the use of mtDNA coupled to HRM analysis to establish the presence and distribution of *Lycalopex* species in Chile and South America. This work detects the bases for further studies of the distribution of these species in Chile, to understand their movement flows in different geographical areas and their adaptation to diverse environments and ecosystems.

## CONFLICT OF INTEREST

The authors have no conflict of interest to declare.

## AUTHORS CONTRIBUTIONS

Leonardo Anabalón: developed the experimental design techniques and elaborated the manuscript; Francisco Encina: developed the experimental design and elaborated the manuscript; Pamela Sanchez: designed the sample and involved in field activities; Jaime Solano: involved in technical development and laboratory work; Felipe Benavente: involved in analysis of sequences; Flavio Olivares; involved in field activity and sampling; Carlos Oberti; involved in handwritten elaboration; Basilio Guiñez: provided the taxonomy control samples; Rolando Vega; elaborated the manuscript and involved in analysis of sequences.

## Data Availability

DNA sequences: Genbank accession AF028151.1.

## References

[ece35230-bib-0001] Behrens‐Chapuis, S. , Malewski, T. , Suchecka, E. , Geiger, M. F. , Herder, F. , & Bogdanowicz, W. (2018). Discriminating European cyprinid specimens by barcode high‐resolution melting analysis (Bar‐HRM). Fisheries Research, 204, 61–73.

[ece35230-bib-0002] Bulinski, J. , & Mcarthur, C. (2000). Spatial distribution of browsing damage and mammalian herbivores in Tasmanian eucalypt plantations. Australian Forestry, 63, 27–33. 10.1080/00049158.2000.10674810

[ece35230-bib-0003] Celis‐Diez, J. L. , Ippi, S. , Charrier, A. , & Garín, C. (2011). Fauna de los bosques templados de Chile. Guía de campo de los vertebrados terrestres. Concepción, Chile: Corporación Chilena de la Madera.

[ece35230-bib-0004] Chaves, P. B. , Graeff, V. G. , Lion, M. B. , Oliveira, L. R. , & Eizirik, E. (2012). DNA barcoding meets molecular scatology: Short mtDNA sequences for standardized species assignment of carnivore noninvasive samples. Molecular Ecology Resources, 12(1), 18–35. 21883979 10.1111/j.1755-0998.2011.03056.x

[ece35230-bib-0005] Cofré, H. , & Marquet, P. (1999). Conservation status, rarity, and geographic priorities for conservation of Chilean mammals: An assessment. Biological Conservation, 88, 53–68.

[ece35230-bib-0006] Cowan, A. F. , & Elkins, K. M. (2018). Detection and Identification of Psilocybe cubensis DNA using a real time polymerase chain reaction high resolution melt assay. Journal of Forensic Sciences, 63(5), 1500. 29194645 10.1111/1556-4029.13714

[ece35230-bib-0008] Davison, A. , Birks, J. D. S. , Brookes, R. C. , Braithwait, T. C. , & Messenger, J. E. (2002). On the origin of faeces: morphological versus molecular methods for surveying rare carnivores from their scats. Journal of Zoology, 257, 141–143.

[ece35230-bib-0007] D'elía, G. , Ortloff, A. , Sánchez, P. , Guiñez, B. , & Varas, V. (2013). A new geographic record of the endangered Darwin's fox *Lycalopex fulvipes* (Carnivora: Canidae): Filling the distributional gap. Revista Chilena De Historia Natural, 86(4), 485–488. 10.4067/S0716-078X2013000400010

[ece35230-bib-0009] Deshpande, K. , Villarreal, M. , & Mills, D. K. (2016). Improved DNA profiles from aged horse feces using pressure cycling technology. Conservation Genetic Resources, 8(4), 487–495. 10.1007/s12686-016-0572-5

[ece35230-bib-0010] Farrell, L. E. , Roman, J. , & Sunquist, M. E. (2000). Dietary separation of sympatric carnivores identified by molecular analysis of scats. Molecular Ecology, 9(10), 1583–1590. 11050553 10.1046/j.1365-294x.2000.01037.x

[ece35230-bib-0011] Fernandes, C. A. , Ginja, C. , Pereira, I. , Bruford, M. W. , & Santos, R. M. (2008). Species‐specific mitochondrial DNA markers for identification of noninvasive samples from sympatric carnivores in the Iberian Peninsula. Conservation Genetics, 9, 681–690.

[ece35230-bib-0012] Fernandes, T. J. R. , Costa, J. , Oliveira, M. B. P. P. , & Mafra, I. (2017). DNA barcoding coupled to HRM analysis as a new and simple tool for the authentication of Gadidae fish species. Food Chemistry, 230, 49–57. 10.1016/j.foodchem.2017.03.015 28407939

[ece35230-bib-0013] Gese, E. M. (2004). Survey and census techniques for Canids In Sillero‐Zubiri C., Hoffmann M. & Macdonald D. W. (Eds.), Canids, foxes, wolves, jackals and dogs: Status survey and conservation action plan (pp. 273–279). Gland, Switzerland: IUCN‐The World Conservation Union.

[ece35230-bib-0014] Hansen, M. , & Jacobsen, L. (2006). Identification of mustelid species: Otter (Lutra lutra), American mink (Mustela vison) and polecat (Mustela putorius), by analysis of DNA from faecal samples. Journal of Zoology, 247, 177–181. 10.1111/j.1469-7998.1999.tb00981.x

[ece35230-bib-0015] Harrison, R. L. (2002). Evaluation of microscopic and macroscopic methods to identify felid hair. Wildlife Society Bulletin, 30, 412–419.

[ece35230-bib-0017] Jeon, H. B. , Anderson, D. , Won, H. , Lim, H. , & Suk, H. Y. (2017). Taxonomic characterization of Tanakia species (Acheilognathidae) using DNA barcoding analyses. Mitochondrial DNA A DNA Mapping, Sequencing and Analysis, 9, 1–10. 10.1080/24701394.2017.1398746 29117773

[ece35230-bib-0018] Johnson, M. K. , Aldred, D. R. , Clinite, E. W. , & Kutilek, M. J. (1981). Biochemical identification of bobcat scats (pp. 92–96). Proc. Bobcat Res. Conf. Nat. Wildl. Fed. Sci. and Tech. Ser., 6.

[ece35230-bib-0019] Kanthaswamy, S. , Premasuthan, A. , Ng, J. , Satkoski, J. , & Goyal, V. (2012). Quantitative real‐time PCR (qPCR) assay for human–dog–cat species identification and nuclear DNA quantification. Forensic Science International: Genetics, 6(2), 290–295. 10.1016/j.fsigen.2011.06.005 21764401

[ece35230-bib-0020] Major, M. , Johnson, M. K. , Davis, W. S. , & Kellog, T. F. (1980). Identifying scats by recovery of bile acids. Journal of Wildlife Management, 44, 290–293.

[ece35230-bib-0021] Medel, R. G. , & Jaksic, F. M. (1988). Ecología de los cánidos sudamericanos: Una revisión. Revista Chilena De Historia Natural, 61, 67–79.

[ece35230-bib-0022] Muñoz‐Pedreros, A. , Yañez, J. , Norambuena, H. V. , & Zuñiga, A. (2017). Diet, dietary selectivity and density of South American grey fox, *Lycalopex griseus*, in Central Chile. Integrative Zoology, 13, 46–57. 10.1111/1749-4877.12260 28262006

[ece35230-bib-0023] Ochoa, M. (2011). Relações filogenéticas entre espécies do gênero Lycalopex (Mammalia, Canidae) inferidas com o uso de marcadores do DNA mitocondrial. Dissertação de mestrado. Pontifícia Universidade Católica do Rio Grande do Sul, Programa de Pós‐graduação em Zoología, Porto Alegre, RS, Brazil.

[ece35230-bib-0024] Onorato, D. , White, C. , Zager, P. , & Waits, L. P. (2006). Detection of predator presence at elk mortality sites using mtDNA analysis of hair and scat samples. Wildlife Society Bulletin, 34, 815–820.

[ece35230-bib-0025] Quinga, M. G. (2012). Estandarización de un protocolo para la extracción de ADN de muestras fecales de lobo de paramo (Lycalopex culpaeus). (Bachelor's degree Thesis), Departamento de Ciencias, Escuela Politécnica del Ejército, Sangolqui, Ecuador.

[ece35230-bib-0026] Ramón‐Laca, A. , Soriano, L. , Gleeson, D. , & Godoy, J. A. (2015). A simple and effective method for obtaining mammal DNA from faeces. Wildlife Biology, 21, 195–203. 10.2981/wlb.00096

[ece35230-bib-0027] Ray, J. , & Sunquist, M. (2001). Trophic relations in a community of African rainforest carnivores. Oecologia, 127(3), 395–408. 10.1007/s004420000604 28547110

[ece35230-bib-0028] Reed, G. H. , & Wittwer, C. T. (2004). Sensitivity and specificity of single‐nucleotide polymorphism scanning by high‐resolution melting analysis. Clinical Chemistry, 50, 1748–1754. 15308590 10.1373/clinchem.2003.029751

[ece35230-bib-0016] Solano, J. , Anabalón, L. , Figueroa, S. , Lizama, C. , Chávez-Reyes, L. , & Gangitano, D. (2019). Psychedelic fungus (Psilocybe sp.) authentication in a case of illegal drug T traffic: Sporological, molecular analysis and identification of the psychoactive substance. Science and Justice, 59, 102–108. 30654963 10.1016/j.scijus.2018.08.005

[ece35230-bib-0029] Taberlet, P. , Waits, L. P. , & Luikart, G. (1999). Noninvasive genetic sampling: Look before you leap. Trends in Ecology and Evolution, 14, 323–327. 10407432 10.1016/s0169-5347(99)01637-7

[ece35230-bib-0030] Tchaicka, L. , Ochotorenas de Freitas, T. R. , Bager, A. , Vidal, S. L. , Lucherini, M. , Iriarte, A. , … Eizirik, E. (2016). Molecular assessment of the phylogeny and biogeography of a recently diversified endemic group of South American canids (Mammalia: Carnivora: Canidae). Genetics and Molecular Biology, 39(3), 442–451. 27560989 10.1590/1678-4685-GMB-2015-0189 PMC5004827

[ece35230-bib-0031] Tende, T. , Hansson, B. , Ottosson, U. , & Bensch, S. (2014). Evaluating preservation medium for the storage of DNA in African lion *Panthera leo* faecal samples. Current Zoology, 60(3), 351–358. 10.1093/czoolo/60.3.351

[ece35230-bib-0032] Tindall, E. A. , Petersen, D. C. , Woodbridge, P. , Schipany, K. , & Hayes, V. M. (2009). Assessing high‐resolution melt curve analysis for accurate detection of gene variants in complex DNA fragments. Human Mutation, 30(6), 876–883. 19280649 10.1002/humu.20919

[ece35230-bib-0033] Torés, N. (2007). Dieta estival del culpeo (Pseudalopex culpaeus, Molina 1782) en Nevados de Chillán, Centro‐sur de Chile. (Bachelor's degree Thesis), Facultad de Ciencias Veterinarias, Universidad Austral de Chile, Valdivia, Chile.

[ece35230-bib-0034] Vossen, R. H. , Aten, E. , Roos, A. , & den Dunnen, J. T. (2009). High‐resolution melting analysis (HRMA): More than just sequence variant screening. Human Mutation, 30, 860–866. 10.1002/humu.21019 19418555

[ece35230-bib-0035] Wang, X. , Xu, G. , Liu, C. , Huang, L. , Zhao, B. , Ren, X. , … Liu, S. (2016). Development of deft amplification refractory mutation sequencing system (ARMSS) for discriminating Pilos antler based on a short cytochrome b (Cytb) gene. Mitochondrial DNA A DNA Mapping, Sequencing and Analysis, 27(2), 1332–1335. 10.3109/19401736.2014.945578 25090397

[ece35230-bib-0036] Wittwer, C. T. (2009). High‐resolution DNA melting analysis: Advancements and limitations. Human Mutation, 30, 857–859. 19479960 10.1002/humu.20951

[ece35230-bib-0037] Yacoub, H. A. , Fathi, M. M. , & Mahmoud, W. M. (2013). DNA barcode through cytochrome b gene information of mtDNA in native chicken strains. Mitochondrial DNA, 24(5), 528–537. 10.3109/19401736.2013.770489 23464748

[ece35230-bib-0038] Ye, J. , Coulouris, G. , Zaretskaya, I. , Cutcutache, I. , Rozen, S. , & Madden, T. L. (2012). Primer-BLAST: a tool to design target-specific primers for polymerase chain reaction. BMC Bioinformatics, 13, 134 10.1186/1471-2105-13-134 22708584 PMC3412702

[ece35230-bib-0039] Zunino, G. E. , Vaccaro, O. B. , Canevari, M. , & Gardner, A. L. (1995). Taxonomy of the genus *Lycalopex* (Carnivora: Canidae) in Argentina. Proceedings of the Biological Society of Washington, 108(4), 729–747.

